# The influence of active carbon contaminants on the ozonation mechanism interpretation

**DOI:** 10.1038/s41598-021-89510-y

**Published:** 2021-05-11

**Authors:** Lilla Fijołek, Joanna Świetlik, Marcin Frankowski

**Affiliations:** Department of Analytical and Environmental Chemistry, Faculty of Chemistry, Adam Mickiewicz University Poznan, Uniwersytetu Poznańskiego 8, 61-614 Poznań, Poland

**Keywords:** Environmental monitoring, Pollution remediation, Environmental impact, Natural hazards

## Abstract

In water treatment technology, activated carbons are used primarily as sorbents to remove organic impurities, mainly natural organic matter, but also as catalysts in the ozonation process. Commercially available activated carbons are usually contaminated with mineral substances, classified into two main groups: alkali metals (Ca, Na, K, Li, Mg) and multivalent metals (Al, Fe, Ti, Si). The presence of impurities on the carbon surface significantly affects the pH_pzc_ values determined for raw and ozonated carbon as well as their acidity and alkalinity. The scale of the observed changes strongly depends on the pH of the ozonated system, which is related to the diffusion of impurities from the carbon to the solution. In an acidic environment (pH 2.5 in this work), the ozone molecule is relatively stable, yet active carbon causes its decomposition. This is the first report that indirectly indicates that contaminants on the surface of activated carbon (multivalent elements) contribute to the breakdown of ozone towards radicals, while the process of ozone decomposition by purified carbons does not follow the radical path in bulk solution. Carbon impurities also change the distribution of the reaction products formed by organic pollutants ozonation, which additionally confirms the radical process. The study showed that the use of unpurified activated carbon in the ozonation of succinic acid (SA) leads to the formation of a relatively large amount of oxalic acid (OA), which is a product of radical SA degradation. On the other hand, in solutions with purified carbon, the amount of OA generated is negligible.

## Introduction

Activated carbons are used in water treatment technology primarily as sorbents to remove organic impurities, mainly natural organic matter^1–6^, but they can also be a catalyst in the ozonation processes^7–19^. In solution, ozone decomposes with formation of hydroxyl radicals, and the process is strongly pH dependant. The lower pH, the more stable ozone molecule is. Oxidation potential of hydroxyl radical is however much higher than of the ozone molecule, and therefore ^•^OH are responsible for oxidation of most organic contaminants. For this reason, ozonation processes are targeted at ozone decomposition and catalysts are used to increase their efficiency. Activated carbons have ability to catalyse oxidation of organic compounds in ozonation processes^8,9,12,20–23^. Basic groups are considered as active centres for O_3_ decomposition by the carbon surface^12,20,23,24^, but there are still doubts about the mechanism of this reaction^25–27^. The main question is: whether ozone decomposed with formation of hydroxyl radicals on the surface of carbons, or the catalytic reaction proceed in the solution^21,22,25–28^. To explain this, it is extremely important to precisely define the properties of carbon surfaces. Basicity and acidity of carbon are determined in reactions with an acid/base after the equilibrium time. Such measurements are subjected to errors especially when elements that can change pH can be find in solution. In the previous work we showed that the mineral matter of unpurified activated carbons contains, in addition to alkali metals, also multivalent metals. The proportions of these groups of elements in the tested carbons are very different^29^. This implies that impurities, especially in alkali metals form, can influence on measurement of acidity and basicity. Recently, Nowicki^30^ indicated that the treatment of raw brown coal activated carbon with an HCl can lead to a change in the acidity and basicity of activated carbon. Similar observation was done by Bazan‑Wozniak et al.^31^, who noticed that purification of activated bio-carbon samples with water and with 5% HCl lead to decrease in basicity and increase in acidity of tested materials. The sorption properties toward iodine and methylene blue also increase after purification^31^. However, to date, no direct evidence has been presented in the literature to confirm the changes in the acidity and alkalinity of unpurified carbons during the ozonation/oxidation processes. Knowledge of the course and scale of this phenomenon is very important because properly defined basic/acid groups are necessary for the interpretation of the reaction mechanism.

Another important parameter characterizing activated carbons, but also other catalysts used in the ozonation process, is the point of zero charge (pHpzc), generally described as the pH at which the net charge of the total material surface is equal to zero. Knowledge of this parameter allows to predict the sorption properties of the tested carbons. The surface chemistry of activated carbons depends mainly on the surface groups containing oxygen. Acid groups on the carbon surface determine the surface charge. The dissociation of these groups will lead to the formation of a negative surface charge. These groups are therefore Bronsted acid centres. The surface charge of activated carbons depends on the pH of the solution^32^. At pH < pH_pzc_, the carbon surface will assume a positive surface charge, while when the pH of the solution is higher than pH_pzc_, the carbon has a negative charge. Hence, at pH < pH_pzc_ anion adsorption will occur on the carbon surface, and at pH > pH_pzc_, cations will be adsorbed. Therefore, the correct determination of the pH_pzc_ parameter is essential when considering the mechanism of the conducted reactions. Since unpurified carbons contain mainly alkaline impurities, their pH_*pzc*_ values will change during the purification process. Indirectly, the influence of mineral matter contained in the activated carbon ash on the pH_*pzc*_ value was indicated by Montes-Moran et al.^33^. During carbon washing with water, the pH_*pzc*_ decreased from 10.6 to 9.8, and the ash content dropped from 6.0 to 4.7%^33^. Significant reduction of pH_*pzc*_ value after carbon purification was also reported by Nawrocki and Fijołek^29^ and Nowicki^30^. However, despite numerous examples of the use of unpurified^12–15^ or only water-washed^16–19^ activated carbon in ozonation processes described in the literature, no direct evidence has been published so far that the pHpzc value of activated carbon may change during ozonation. Most studies do not analyse the amount and type of impurities on the carbon surface and their impact on the course and efficiency of pollutants removal in ozonation processes. However, there are some evidence that carbon nanotubes impurities can enhance redox properties of carbon and improve electrooxidation of organics^34,35^. In turn, iron impurity of activated carbons can enhance phenol removal in catalytic wet peroxidation^36^. In our previous work^29^, it was shown that the mineral matter presence in activated carbon enhances the ozone decomposition. Homogeneous catalysis of ozonation processes by metal ions are known as well^25–27,37^, therefore it can be expected that carbon contaminants will have an impact on the ozonation process. However, the literature describing this phenomenon in more detail is limited. Rivera-Utrilla and Sanchez-Polo^20^ observed a decrease in the catalytic activity of carbons after their purification from mineral impurities in the process of 1,2,3-naphthalenetrisulfonic acid ozonation.

The aim of this study was to show that the determined values of selected physico-chemical parameters of unpurified activated carbons may change during the ozonation and differ depending on the pH of the process. The research analysed whether the processes involving active carbons take place on the carbon surface or in the bulk solution as well as what is the role of carbon contamination in the process. The relationships described in the paper show how important is proper preparation of the catalyst before its use in the catalytic process. The results of the presented research have shown that the use of unpurified activated carbon in ozonation processes leads to the observation of “false” catalytic effects. This has been shown by the ozonation of succinic acid (SA) as an example. One of the main products of succinic acid oxidation is an oxalic acid (OA), hence its presence in the solution is an indirect evidence of •OH radicals’ formation in the tested systems. In this study, during the ozonation process in the presence of unpurified carbons, significant concentrations of OA in the solution were determined, which proves the radical path of SA oxidation. The radical course of the process in the presence of unpurified activated carbon was confirmed in experiments with tert-butyl alcohol (TBA).

## Materials and methods

### Activated carbons

The following carbons were used in the tests: F300 (Filtrasorb 300; Chemviron Carbon), F400 (Filtrasorb 400; Chemviron Carbon), 830 W (GAC 830 W- Norit), Silcarbon (Silcarbon S1020; Acivhole), Aquasorb (Aquasorb 6300 8 × 30—Jacobi Carbons), DGF (DGF AX 8 × 30/65; Carbotech AC), 830S (GAC 830Supra—Norit). All tested activated carbons were thoroughly characterized in^29^.

### Ozonation of activated carbons

Unpurified carbons were ozonated at room temperature in unbuffered high purity water (Millipore). The pH was adjusted to 2.5 or 5.0 with HCl. In each experiment, 200 ml of high purity water at a specified pH was introduced into a 250 ml glass reactor. At the same time, 0.5 g of activated carbon was introduced into the reactor. Ozone was produced from pure oxygen using a crystal ozonator (Canada). Water was saturated with ozone for a period of 20 min. The ozonation process for each of the activated carbon was repeated six times. The samples obtained in replicates were combined and then dried at 120 °C for a period of 4 h.

### Activated carbons purification

The carbons were purified according to the procedure described in^29^. Briefly: the carbons were extracted with HCl (azeotropic aqueous solution) for 5 h followed by high purity water (Millipore) for a further 5 h. The content of elements in the solutions after extraction was measured with ICP-OES (Varian ICP-OES Vista-MPX) Compositions of post-purified extracts of the tested activated carbons were given in full detail in^29^.

### Ozonation of succinic acid

The process of succinic acid (Aldrich) ozonation was carried out at room temperature in unbuffered high purity water (Millipore). After addition of succinic acid (180 mg/l; 1 mM) to the water, the pH of the solution was adjusted to 2.5 with HCl. In each experiment, 200 ml of succinic acid solution and the appropriate amount of activated carbon were introduced into a 250 ml glass reactor. Ozone was produced from pure oxygen using a crystal ozonator (Canada) and introduced into the system continuously for 60 min. During this period, samples were taken at designated intervals to determine the remaining content of unoxidized acid and the amount of oxalic acid formed during ozonation. Ozone remaining in the water was dispersed by the immediate addition of Na_2_SO_3_. Each series of experiments was done three times.

### Determination of pH_pzc_

The determination of the pH_pzc_ of the carbons was carried out as follows: 100 cm^3^ of 0.01 M NaCl solution was placed in a plastic vessel. The pH of the solution was adjusted to a value between 3 and 11 with 0.1 M HCl or 0.1 M NaOH. Then, 0.3 g (+ /- 0.005 g) of activated carbon was added and the vessels were tightly closed. The final pH was measured after 24 h of agitation at room temperature. The pH_pzc_ value determined the intersection of the pH_final_ vs. pH_inital_ and pH_initial_** = **pH_final_ curves.

### Determination of the acidity and alkalinity of activated carbons

To determine the acidity and alkalinity of activated carbons’ the surface, 25 mM NaOH and 25 mM HCl were prepared. 50 ml of base/acid solution was added to the plastic vessels and 0.4 g of the tested carbon weighed to an accuracy of ± 0.005 g was added. The vessels were then sealed and allowed to equilibrate at room temperature for 24 h. After this time, the solutions were decanted and the amount of remaining base/acid was determined by titration with 25 mM HCl and 25 mM NaOH, respectively.

### Carboxylic acid determination

The concentration of succinic acid and oxalic acid was measured by ion exclusion chromatography using a Waters 2690 Chromatograph equipped with a UV–Vis detector (Waters 2487 Dual λ) and an Aminex HPX-87 analytical column. The mobile phase was water acidified to pH 2 with H_2_SO_4_. The mobile phase flow was 0.6 ml/min. Detection was carried out at λ = 210 nm. Limit of quantification (LOQ) for SA was 1 mg/l (5.5 µM) and for OA: 8 µg/l (88 nM); in both cases RSD was below 3%.

## Results and discussion

### Mineral contamination versus physical and chemical parameters of activated carbons

Commercially available activated carbons are more or less contaminated with mineral matter, which mainly consist of alkaline (Ca, K, Na, Li, Mg) and multivalent (Ti, Al, Fe, Si) elements in different ratios that are presented in^29^. The unpurified activated carbons tested are characterized by the highest pH_*pzc*_ values (Table 1). Removal of mineral matter from surfaces causes a significant decrease in pH_*pzc*_ values for all tested carbons (Table 1), and confirms the earlier observations^29–31^. Ozonation of unpurified carbons caused a decrease in the pH_*pzc*_ value as well. Purified, non-ozonated activated carbons have lower pH_*pzc*_ values than unpurified carbons ozonated at pH 2.5 and 5.0 (Table 1). These results clearly show a significant influence of impurities on pH_*pzc*_ determination, which will be discussed further.

**Table 1:** Changes in pH_*pzc*_ values due to purification and ozonation of activated carbons: unpurified non-ozonated, unpurified ozonated (pH 2.5 and 5) and purified non-ozonated (E) [in µeq/g].

	F300	F400	830 W	830S	Silcarbon	Aquasorb	DGF	Ref
Unpurified/non-ozonated	10.0	9.9	10.0	10.9	10.3	10.3	8.0	^22^
Unpurified/ozonated 2.5	9.2	7.7	7.4	9.0	9.2	7.6	6.8	
Unpurified/ozonated 5.0	9.3	9.8	8.7	10.0	10.2	8.9	7.5	
Purified/non-ozonated	8.9	7.1	7.4	8.5	8.8	6.9	6.0	^22^

Moreno-Castilla et al.^38^ found that the extraction of activated carbon with acid does not change the amount of oxygen functional groups on the carbon surface^38^, therefore, in our work the carbons were purified with non-oxidizing acid (HCl) to eliminate oxidation of the carbon surface during their purification. On the other hand, ozonation of carbons contributes to the oxidation of their surface, on which acid functional groups are formed. However, if the changes in pH_*pzc*_ were caused only by the oxidation of carbons, then one would expect the same pH_*pzc*_ after ozonation of activated carbons at different pH values. However, ozonation of unpurified commercial activated carbons at pH 2.5 always leads to a lower pH_*pzc*_ compared to the process carried out at pH 5.0 (Table 1), which is related to the higher solubility of the mineral matter at a lower pH. Measurements of pH_pzc_ are carried out in electrolyte solutions, so it would seem that impurities should not affect the result. However, the results obtained within this work indicate a significant influence of impurities on the determination of the surface properties of the carbons. Due to the fact that the equilibrium time for which activated carbons are left, it is up to 48 h^39^, alkaline impurities diffuse from the inside of the carbon structure into the solution, influencing the determination of the pH_*pzc*_ value^29–31^. Consequently, the characteristics of unpurified activated carbons and their subsequent use in the ozonation process are subject to errors, as the determined pH_*pzc*_ values ​​change during the ozonation process (Table 1). Due to the presence of impurities, changes in the acidity/alkalinity of the tested carbons can also be expected. The acidity and alkalinity of the surfaces of the analysed materials were determined in samples of unpurified, purified and unpurified ozonated carbon at pH 2.5 and 5.0. The results are shown in Tables 2 and 3.

**Table 2:** Acidity (in µeq/g) of activated carbons: unpurified, non-ozonated, unpurified ozonated (at pH 2.5 and 5.0) and purified non-ozonated.

	F300	F400	830 W	830S	Silcarbon	Aquasorb	DGF
Unpurified/non-ozonated	169	212	198	227	132	235	352
Unpurified/ozonated 2.5	245	241	194	246	209	250	346
Unpurified/ozonated 5.0	278	340	216	295	256	350	429
Purified/non-ozonated	406	506	319	347	251	361	500

**Table 3:** Alkalinity (in µeq/g) of activated carbons: unpurified, non-ozonated, unpurified ozonated (at pH 2.5 and 5.0) and purified non-ozonated.

	F300	F400	830 W	830S	Silcarbon	Aquasorb	DGF
Unpurified/non-ozonated	606	616	549	581	804	888	792
Unpurified/ozonated 2.5	588	533	489	553	618	669	707
Unpurified/ozonated 5.0	489	425	476	504	528	508	646
Purified/non-ozonated	497	460	319	493	478	375	458

Remove of impurities from carbons in the extraction process causes changes in the value of its acidity and alkalinity^30,31^. The most significant changes in acidity (increase) and alkalinity (decrease) are observed for purified carbons, which is undoubtedly associated with the removal of mineral matter from their surface. On the other hand, the ozonation of unpurified carbon at pH 2.5 leads to greater changes than the ozonation at pH 5.0. This is due to the previously described increasing solubility of mineral matter of carbons in an acidic environment. The obtained results indicate that the result of the titrations will mainly depend on the content of impurities in the activated carbon. The basicity determinations were carried out by reacting the tested carbons with an acid. When unpurified carbons were used, then not only surface groups of basic nature will react with the acid, but also alkaline impurities present on the surface of the carbons. Consequently, this procedure will lead to an erroneous (overestimated) result of the alkalinity determination, and the magnitude of the error will increase as the pH decreases. During ozonation, the surface of activated carbons oxidizes^40^, and therefore the acidity of carbons should also increase. However, if the increase in acidity was only the result of the ozonation process, then ozonation at both pH 2.5 and 5.0 should lead to equal changes in the value of this parameter. As can be seen from the experimental data presented in Tables 2 and 3, both acidity and alkalinity are different depending on the pH of the ozonated solution. It proves that the process of diffusion of mineral impurities from the surface of the analysed materials occurred.

In addition, these results are evidence of the significant impact of the impurities presence on the correct measurements of surface properties of activated carbons. Moreover, they also indicate on the possibility to make a catalytic reaction mechanism misinterpretation when unpurified activated carbons are used in this process. Such errors will be particularly significant when high amounts of carbon will be used. The obtained results clearly indicate on significant role of impurities in determination of the acid–base properties of commercially available activated carbons as well as the possibility of changes the value of this parameter during ozonation.

### Impact of activated carbon impurities on the ozonation process

Organic compounds, especially with electron withdriving substituents at the aromatic ring, can be easily removed in self-enhanced ozonation processes at low pH^41,42^. Succinic acid does not possess aromatic ring, therefore self-enhance ozonation reaction could not be expected. Furthermore, during succinic acid (SA) ozonation, oxalic acid may be formed as one of the reaction by-products. Hence, the OA detection in the ozonated SA solution can be taken as evidence of degradation of succinic acid. Both of these relationships determined that SA was selected as the model compound in this study. Ozonation processes were carried out with unbuffered, high purity water, whose pH was adjusted to 2.5 with HCl. It is known that chlorides inhibit radical processes by reaction with hydroxyl radicals^42,43^. Additionally, at low pH (2.5), the ozone molecule is relatively stable and does not easy decompose with the formation of hydroxyl radicals. The ^·^OH oxidation rate constant of SA (3.1 × 10^8^ M^−1^ s^−1^) is much higher than its ozonation rate constant (less than 0.03 M^−1^ s^−1^) at acidic pH^44^. For those reasons, low succinic acid removal efficiency, and hence low oxalic acid generation, can be expected.

**Figure 1:**
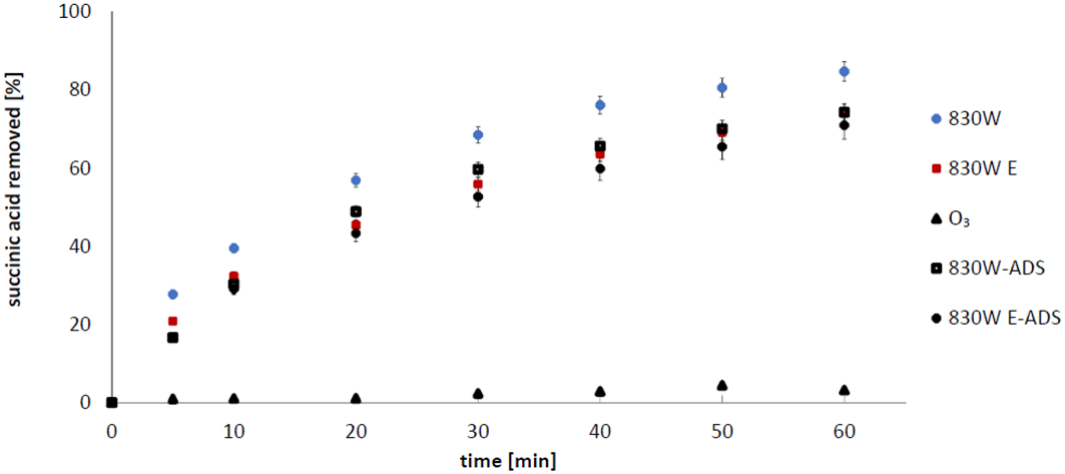
The process of removing succinic acid (1 mM) on 830 W carbon (pH 2.5; 2 g/200 ml): 830 W E-ADS—adsorption on purified carbon; 830 W-ADS—adsorption on unpurified carbon, 830 W—ozonation with unpurified carbon; 830 W E—ozonation with purified activated carbon; O_3_—ozonation without activated carbon addition.

**Figure 2:**
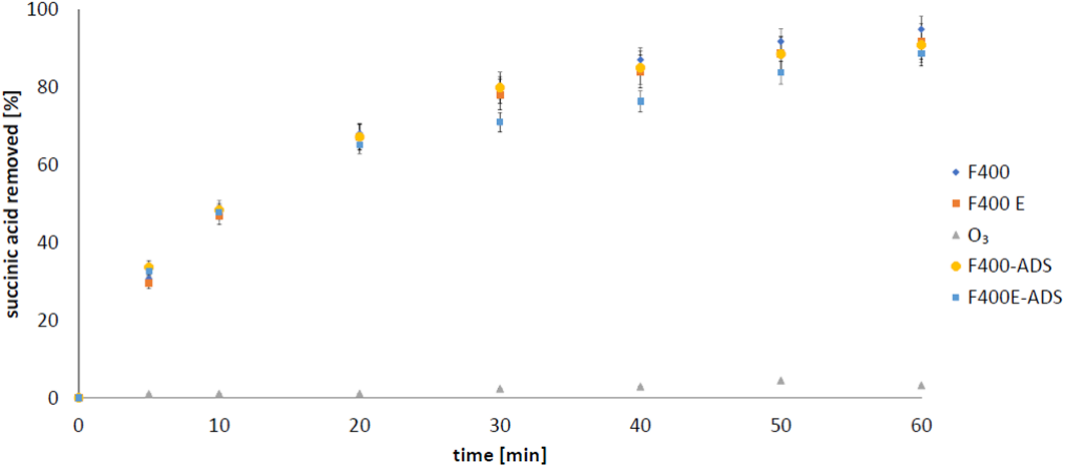
The process of removing succinic acid (1 mM) on F400 carbon (pH 2.5; 2 g/200): F400E-ADS—adsorption on purified carbon; F400-ADS—adsorption on unpurified carbon, F400—ozonation with unpurified carbon; F400 E—ozonation with purified activated carbon; O_3_—ozonation without activated carbon addition.

In our previous work^29^, we showed that activated carbons 830 W and F400 are characterized by the highest content of impurities in the form of multivalent metals, that is why they were selected for ozonation processes in this study. In the first stage of research, succinic acid adsorption was carried out on selected carbons. The obtained results are presented in Figs. 1 and 2. During ozonation of succinic acid, 74.3% of SA (mean 136.8 µg/l) was adsorbed in the presence of 830 W (unpurified carbon), and 47.2 µg/l of SA remained in the solution, representing 25.7%. For the same but purified carbon, the adsorption was slightly lower, because 71.0% of SA (130.9 µg/l on average) was removed, and 29.0% (53.5 µg/l) of succinic acid remained in solution (Fig. 1). For carbon F400, the observed adsorption degree was higher compared to 830 W and was 90.9% (on average 170.4 µg/l) adsorbed and 17.12 µg/l (9.1%) of SA remained in solution. In turn, after the extraction of impurities from the F400 carbon, slightly less adsorption occurred—88.7% (165.6 µg/l) of succinic acid was removed, and 21.2 µg/l (11.3%) SA remained in the solution (Fig. 2). It can therefore be stated that the efficiency of succinic acid adsorption on activated carbons is therefore high, and the high degree of succinic acid removal can be connected with this phenomenon.

**Figure 3:**
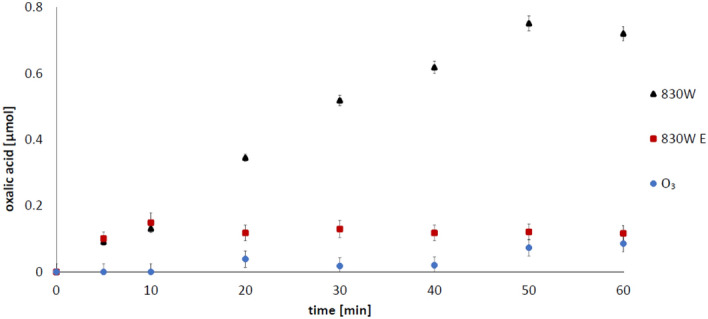
Impact of 830 W carbon impurities on oxalic acid formation at pH 2.5 (2 g/200 ml), 830 W—unpurified carbon; 830 W E—purified carbon, O_3_—ozonation without activated carbon addition. Initial succinic acid concentration 1 mM.

**Figure 4:**
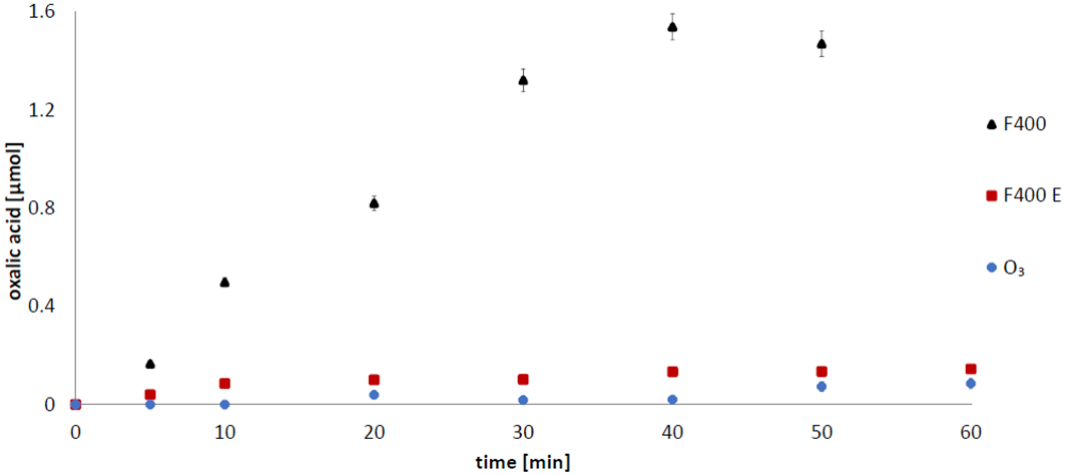
Impact of F400 carbon impurities on the amount of oxalic acid formed at pH 2.5 (2 g/200 ml): F400—unpurifed carbon, F400 E—purified carbon, O_3_—ozonation without activated carbon addition. Initial succinic acid concentration 1 mM.

Oxalic acid (OA) is one of the radical oxidation products of succinic acid. The presence of oxalic acid in the reaction mixture may therefore indirectly indicate the presence of hydroxyl radicals in the ozonated solution. During ozonation of the SA solution in the presence of examined carbons, the OA was detected in all cases. After ozonation of SA in the presence of 830 W, OA was determined in concentrations of 0.754 µM (for unpurified 830 W) and 0.167 µM (for purified 830 W (E)), respectively (Fig. 3). In the presence of F400 carbon, it has been shown that the highest amounts of oxalic acid are formed in the presence of unpurified carbon for which also higher SA adsorption efficiency has been demonstrated (Figs. 1, 2). In the presence of contaminated F400 carbon, an average of 1.538 µM of oxalic acid was formed, while for purified carbon only 0.073 µM was detected (Fig. 4).

**Figure 5:**
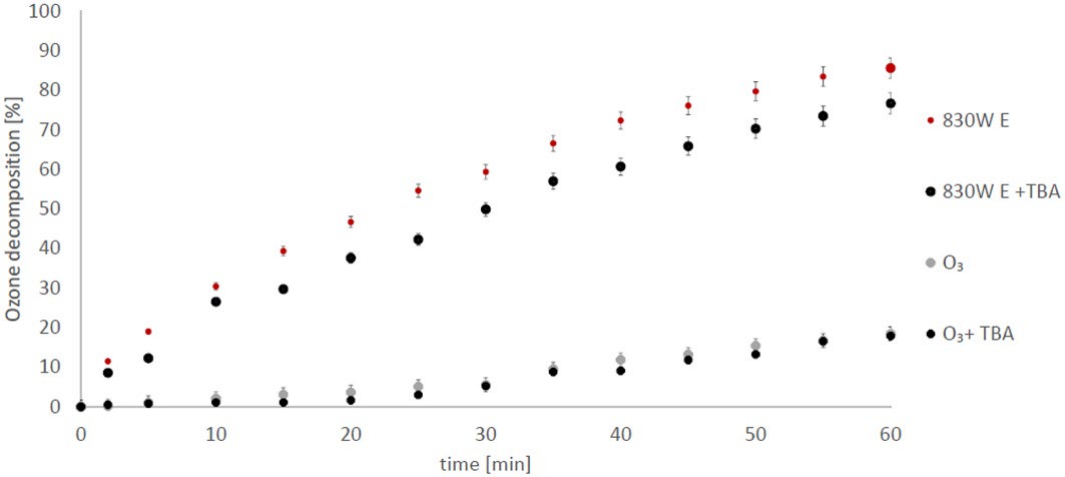
Process of ozone decomposition at pH 2.5 in the presence: 830 W E—purified carbon (0.5 g/200 ml); 830 W E + TBA—purified carbon with TBA addition; O_3_—ozonation without carbon, O_3_ + TBA—ozonation without carbon in the presence of TBA. E—purified carbon).

**Figure 6:**
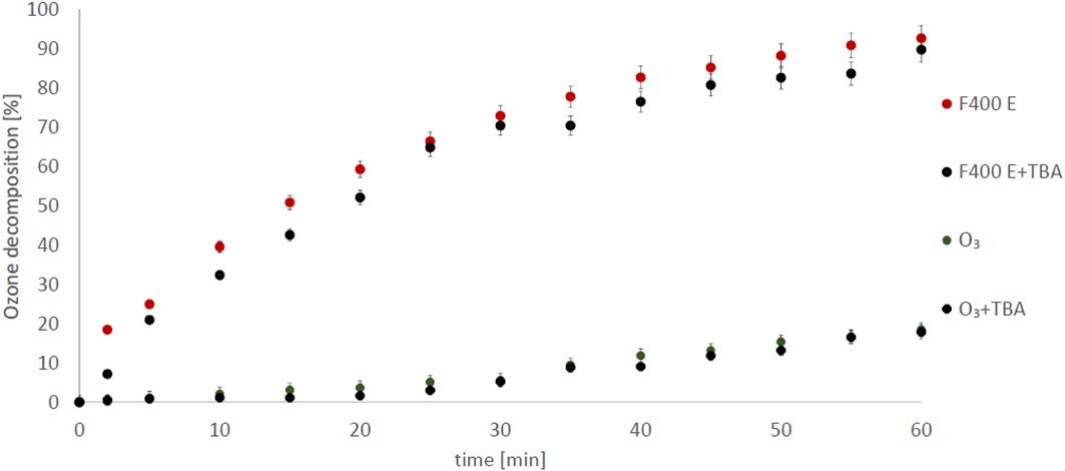
Process of ozone decomposition at pH 2.5 in the presence: F400 E—purified carbon (0.5 g/200 ml); F400 E + TBA—purified carbon with TBA addition; O_3_—ozonation without carbon, O_3_ + TBA—ozonation without carbon in the presence of TBA.

It was speculated that catalytic reactions occur on the carbon surface^20,22^. In this study, the observed efficiency of succinic acid adsorption was the highest in the case of unpurified carbon F400, which, together with the high concentration of oxalic acid found in the reaction mixture, clearly indicates that the degradation reaction of succinic acid occurs on the surface of the activated carbon. Similar results were obtained by Xing et al.^45^, who showed that ozonation of oxalic acid is most efficient in acidic condition, where acid sorption was also the highest. An additional factor that may affect the degree of succinic acid degradation is ozone decomposition on the carbon surface, which may result in the formation of processed product—hydroxyl radicals^46^. As we showed in the previous work^29^, the presence of carbon mineral impurities contributes to the intensification of ozone decomposition. To confirm/exclude the presence of hydroxyl radicals formed on the carbon surface, in this work, ozone decomposition was performed using purified carbons. The pH of the solutions in this case were adjusted with HClO_4_, which has no effect on the radical processes. The obtained results confirm that activated carbon significantly contributes to the ozone decomposition (Figs. 5, 6).

To confirm that ozone decompose on the surface of the carbons, tert-butyl alcohol (TBA) was used in further experiments. TBA reacts very slowly with the ozone molecule (k = 3 × 10–3^47^), but very quickly with the hydroxyl radical (k = 6.3 × 108^48^) and in practice is used to scavenge hydroxyl radicals from solution^28^. If the reaction continues in the presence of TBA, then its efficiency is not related to the presence of hydroxyl radicals in the solution. The experiments presented in Figs. 5 and 6 have shown that, despite the presence of TBA (4 mM) in solution, ozone decomposition in the presence of carbons still occurs. This means that the decomposition of ozone can proceed on the surface of purified activated carbons and no hydroxyl radicals are formed in the bulk solution. These observations stand with accordance with our previous results^28^. Xing et al.^45^, observed similar phenomenon. The authors showed that ozonation of oxalic acid is influenced in small degree by the TBA in the presence of carbon (0.5 g/L). However, the experiments were carried out at a pH 7.0, in which the amount of hydroxyl radicals may be higher (than in acidic condition), at least due to the higher content of ־OH, a known ozone decomposition initiator. In turn Faria et al.^22^ had noticed that TBA presence in ozonated solution have no influence on oxalic acid ozonation in pH 3. This results are with accordance with our observations and indicate that surface reaction play key role in catalytic processes. Regrettably, in those works there are no information about activated carbons purification before use (if any), therefore correlation of catalytic effect with carbons impurities is difficult.

As indicated above, the presence of oxalic acid in the analysed samples can be used as an indicator of the hydroxyl radicals in the system. During ozonation without carbon, succinic acid is practically not degraded. Oxalic acid is also not produced in this process (Figs. 3, 4). During ozonation in the presence of purified carbons, the amount of oxalic acid detected is very low as well. The dominant impurities of the tested activated carbons are Al, Fe, Ti, Si^29^, and as shown above, the carbons purified from those metals do not contribute to the formation of hydroxyl radicals in the bulk solution (Figs. 5, 6). However, it cannot be ruled out that also in this case ozone is decomposed on the carbon surface. Interpretations of the results obtained during these studies are well complemented by the results of the experiments presented in our previous work. We showed that unpurified activated carbons decompose ozone to a greater extent than purified carbons^29^. It can therefore be concluded that multivalent metals, which are the main impurities of the activated carbon, are primarily responsible for the decomposition of ozone towards radicals in the bulk solution. Then, the hydroxyl radicals formed with their participation attack the adsorbed succinic acid molecules, producing the oxidation reaction by-product—oxalic acid. Earlier we showed^49^ that during the ozonation of activated carbons, hydrogen peroxide is generated and its significant amount was detected only at low pH, so as in this study (pH 2.5). It was also shown^29^ that iron ions right after aluminium ions are the dominant impurity of both carbons. Therefore, a possible route of the formation of radicals in the solution is also the process of hydrogen peroxide decomposition by iron ions (Fenton's process), since it is hard to expect that hydrogen peroxide will be decomposed by aluminium ions. H_2_O_2_ could be also decomposed by carbon surface, however, this process is limited in acidic conditions^50^. Moreover, we also checked [^49^—Supplementary Information] that chlorides have no influence on the decomposition of hydrogen peroxide in the presence of activated carbon. The above results confirm our statement that the impurities have an influence on the ozonation reaction. The activity of mineral matter in the ozonation process was partially observed by Rivera-Utrilla and Sanchez-Polo^20^ who, after removing mineral impurities from carbon, observed a lower degree of mineralization of 1,2,3-naphthalenetrisulfonic acid at pH 2.3^20^.

In this study, using OA detection, we confirmed that carbon impurities influence on the ozonation processes leading to the formation of hydroxyl radicals in solution. In addition, it was shown that presence of mineral matter can change the determined values of carbon parameters when it is ozonated at different pH.

## Conclusions

Mechanism of ozonation is strictly related to the chemistry of carbons, pH_pzc_ values can specify sorption properties of activated carbons, while amount of acidic and basic groups indicates the type of catalytic reaction centres. As a consequence, proper determination of those parameters is very important for processes carried out with the use of carbons. In turn, any errors in determining these parameters may lead to significant misinterpretations in determining the reaction paths.

This paper showed, that the presence of impurities on the surface of activated carbons affect the measurements of parameters related with their surface chemistry in the course of ozonation. Purification of activated carbons lead to decrease of pH_pzc_ values by 1.1–3.4 units. At the same time the basicity decreases by 1.2–2.4 times, while acidity increases 1.4–2.4 times.

Carbons impurities can also enhance ozonation efficiency, as was shown by the example of SA degradation confirmed by the presence of OA—succinic acid ozonation by-product. In contrary to purified carbons, the application of unpurified activated carbons lead to generation of significant amounts of oxalic acid. These results indirectly indicate that impurities on the surface of active carbons (i.e., multivalent elements) contribute to the generation of hydroxyl radicals.

It was shown, that the process of ozone decomposition by purified carbons does not lead to the hydroxyl radical formation in the bulk solution. These results were confirmed by the TBA no impact on ozone decomposition processes by purified carbons.

During ozonation in the presence of active carbons at low pH, significant amounts of H_2_O_2_ is formed. Unpurified activated carbons can contain large amount of iron in their structure as well. In case of carbons used in this study even 71–75 µmol of iron ions can be released from 1 g of carbon. As a consequence, •OH radicals are most likely formed in a solution containing carbon impurities (polyvalent elements) during the decomposition of hydrogen peroxide in the Fenton process. Then, the so formed radicals attack SA adsorbed on the carbon surface and oxidize it with the formation of OA.

As shown in this study, proper preparation of activated carbon before catalytic run is very important. Activated carbon impurities can have a significant impact on the process of organic pollutants ozonation, which may lead to observation of “false” catalytic effects and misinterpretation of the obtained results. Nevertheless, the use of unpurified carbons in ozonation processes can enhance the catalytic effect.
